# Fulminant intravascular hemolysis resulting from *Clostridium perfringens* infection

**DOI:** 10.1002/ajh.27511

**Published:** 2024-10-22

**Authors:** Kyo J. P. H. Renshof, Yorick Sandberg, Floor Weerkamp, Barbara J. Bain

**Affiliations:** ^1^ Department of Internal Medicine Maasstad Hospital Rotterdam The Netherlands; ^2^ Department of Clinical Chemistry Maasstad Hospital Rotterdam The Netherlands; ^3^ Centre for Haematology, St Mary's Hospital Campus of Imperial College Faculty of Medicine St Mary's Hospital London UK

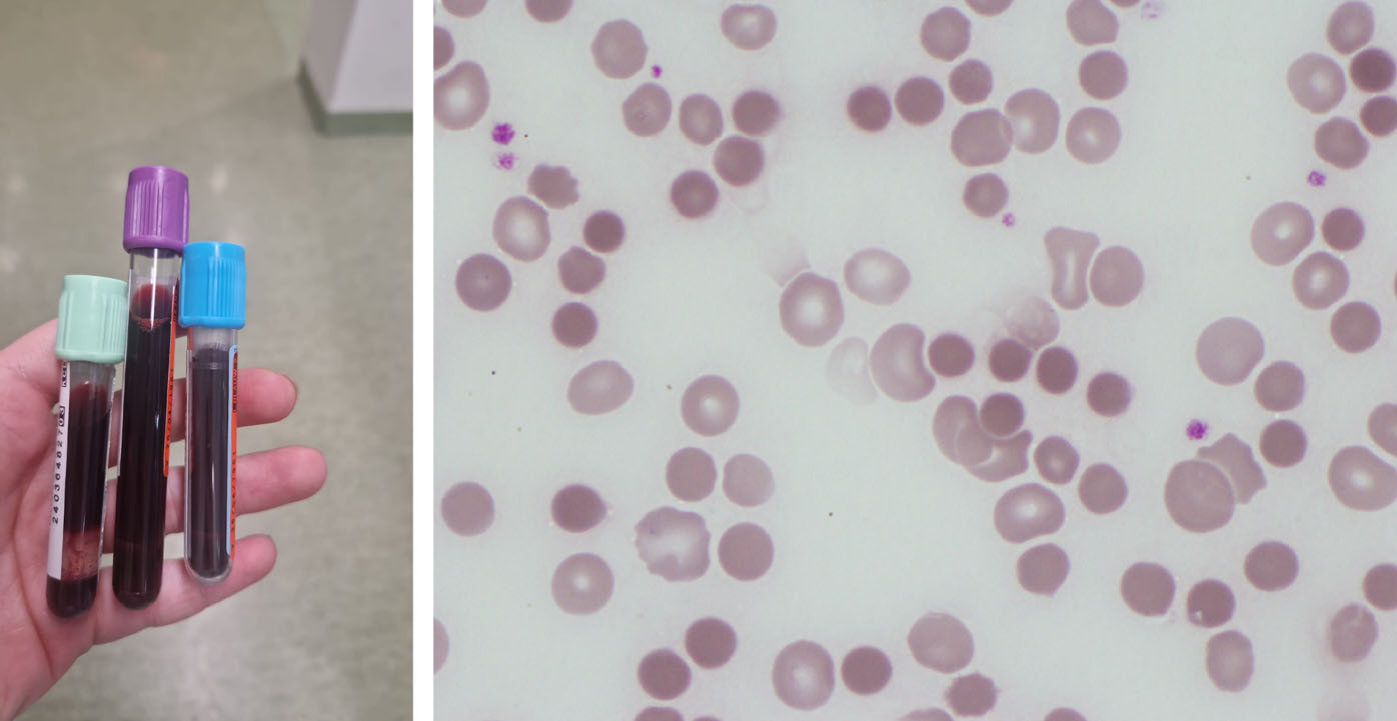

A 70‐year‐old woman with a history of successfully resected pancreatic cancer 10 years ago presented with dyspnea and fever. Laboratory tests showed leukocytosis (white cell count 20.8 × 10^9^/L) and elevated C‐reactive protein (87 mg/L) with normal hemoglobin concentration (Hb) (137 g/L) and platelet count (242 × 10^9^/L). Computed tomography identified a hepatic abscess. The abscess was drained and ceftriaxone‐metronidazole was administered intravenously. Sixteen hours after presentation, the patient's condition deteriorated, with Hb dropping to 50 g/L while the platelet count remained normal. Macroscopically the blood sample appeared dark (left image), and no red blood cells could be separated upon centrifugation. A blood film showed spherocytosis and dehemoglobinized ghost cells (right image, May–Grünwald–Giemsa ×100 objective), indicating acute intravascular hemolysis. Despite early drainage plus antibiotic treatment, and admission to the intensive care unit, the patient died 21 h after initial presentation. Blood and abscess cultures grew *Clostridium perfringens* (metronidazole susceptible).

*C. perfringens* liver abscess and subsequent sepsis with fulminant intravascular hemolysis are rare, but have been previously documented.^1^ Alpha‐toxin secretion induces spherocytosis and hemolysis by disrupting cell membrane integrity via phospholipase activity.^2^ This case emphasizes that despite prompt diagnosis and treatment, this condition can be fatal within hours of initial presentation. A recent case report suggests that toxin‐clearing interventions, following rapid diagnosis, may improve outcome in patients with acute hemolysis due to *C. perfringens* sepsis.^3^ Early treatment requires efficient communication between the laboratory and clinicians when blood samples raise the suspicion of intravascular hemolysis, followed by prompt blood film examination. Spherocytosis and the presence of ghost cells are important in suggesting this particular infection.

## CONFLICT OF INTEREST STATEMENT

The authors declare no conflict of interest.
